# 319. Infectious disease healthcare utilization and outcomes of patients discharged to skilled nursing facilities on parenteral antibiotic therapy

**DOI:** 10.1093/ofid/ofad500.390

**Published:** 2023-11-27

**Authors:** Rosemary C Bailey, Christopher J Crnich, Sally Jolles, Jon P Furuno, Brie Noble, Madeline C Langenstroer

**Affiliations:** UW Health - Madison, WI, Madison, Wisconsin; University of Wisconsin School of Medicine and Public Health, Madison, Wisconsin; University of Wisconsin School of Medicine and Public Health, Madison, Wisconsin; Oregon State University, Portland, Oregon; Mayo Clinic, Scottsdale, Arizona; University of Wisconsin School of Medicine and Public Health, Madison, Wisconsin

## Abstract

**Background:**

Hospitalized patients are frequently discharged to a skilled nursing facility (SNF) for parenteral antibiotic therapy (PAT). While there are studies describing the healthcare utilization and outcomes of individuals receiving post-hospital PAT in non-institutional settings, characterization of the PAT experience among SNF patients remains limited. To address this gap in knowledge, we conducted a retrospective cohort study of hospitalized patients discharged to a SNF on antimicrobial therapy.

**Methods:**

Health records of patients (age ≥18 years) hospitalized in a single hospital who were discharged on systemic antibiotics to a SNF from 1/1/2016 - 12/31/2018 were reviewed. Subjects who received an inpatient ID consultation and were discharged on PAT were included. Information captured included patient demographics, diagnoses, discharge antibiotic regimen and timing, post-discharge encounters, 90-day hospital readmissions, and losses to follow-up. This study was approved by the Institutional Review Board with a waiver for informed consent.

**Results:**

155 patients meeting the inclusion criteria were identified. Post-discharge follow-up with the ID Service was not recommended for 19 patients (12.3%). Patients who followed-up post-discharge had a total of 264 sub-specialty provider (mean: 2.8/patient; range: 0-13) and 170 ID provider (mean: 1.7/patient; range: 0-5) face-to-face encounters within 90 days of discharge. Average time to ID clinic follow-up was 3.2 weeks (range: 1-6 weeks). ID clinic nursing staff conducted 1373 telephone encounters (mean: 13.9/patient; range, 3-40) with SNF nursing staff within 90 days of discharge. The 90-day hospital readmission rate was 37% (36/101 patients) and 47% of readmissions were ID-related. The 90-day mortality rate was 9%; 2 patients died before and 7 after outpatient follow-up was established.

**Conclusion:**

ID clinic follow-up of patients discharged to a SNF on PAT was infrequent, but their care entailed a higher volume of telephone care coordination, and they experienced higher readmission rates relative to patients receiving PAT in ambulatory settings. Further studies to better understand if these findings are driven by patient factors or issues related to SNF care practices are needed.

**Disclosures:**

**All Authors**: No reported disclosures

